# On the Recent Advances in the Theory of Vision

**Published:** 1872-05

**Authors:** H. Helmholtz

**Affiliations:** The Royal Society of London


ART. III.—*On the recent Advances in the Theory of Vision. By* H. HELMHOLTZ, (*of the Royal Society of London.*)

Translated from *Les Annales d' Oculistique*, BY F. W. ABBOTT, M. D.

(*Continued.*)

The theory of color, with all the curious and complicated circumstances which I have just described, was a problem which was insoluble not only by Goethe, but other physicists and physiologists. I myself, likewise, wasted a long time in unavailing efforts, when I finally discovered that a solution of surprising simplicity had already been found and published, at the commencement of this century. It was by the same Thomas Young who took the first step in reading the Egyptian hieroglyphics. He was one of the most profound geniuses who have ever lived; but he had the misfortune to be in advance of his age. His contemporaries admired him without being capable of following the bold flight of his combinations. Hence a quantity of the most important of his ideas remained buried and forgotten in the records of the Royal Society of London, until a new generation made again laboriously and step by step his discoveries, and became convinced of the accuracy of his assertions and demonstrations.

While explaining here the theory of colors established by Young, I will observe that the conclusions which we shall draw from it farther on, relative to the nature of visual sensations, are entirely independent of what this theory presents that is hypothetical.

Thomas Young says that there are in the eye three kinds of nerve fibres, the first of which, excited it matters not how, produce the sensation of red; the second, the sensation of green, the third that of violet. He says, further, that the first are excited the most by the luminous vibrations of the ether whose wave-length is greatest, that the fibres sensitive to green are excited especially by waves of medium length, and that the fibres sensitive to violet are principally excited by rays of the shortest wave-length. Thus, the irritation of the fibres sensitive to red would predominate in the red extremity of the spectrum, and it is for this reason that this extremity appears red to us; farther on an irritation of the fibres sensitive to green would be added, and it is to the mixture of these sensations that we give the name of yellow: in the middle of the spectrum, the irritation of the nerves sensitive to green would dominate over both the others and the sensation of green would be dominant; the mixture of this sensation with that of violet would give blue; finally, at the end of the spectrum which is most refracted, the sensation of violet would dominate.*

We see that the hypothesis of Young is nothing else than a specialisation of the law of the specific energies of the senses, just as much as that the difference between the sensation of light and that of heat depends solely upon the nature of the terminal fibres, optical or sensitive, which are hit by the rays of the sun. According to the hypothesis of Young, the difference between the sensations of color depends upon the relative degree of irritation of the three kinds of fibres of which he supposed the existence. The equal irritation of these diverse fibres gives the sensation of white.

We may therefore explain the phenomena of anerythropsy by supposing that the fibres sensitive to red are incapable of irritation. It is probable that towards the periphery of the retina of the normal eye these fibres sensitive to red are wanting, or are very few in number.

In man and the other mammalia, we do not yet know any anatomical fact which we can bring to the support of this theory of the colors. But Mr. Max Shultze has found in birds and reptiles a structure which evidently hears a relation to this theory. In the eyes of many of these animals, some of the rods of the sensitive retinal layer have a red oily droplet upon their anterior end, the one turned towards the light; other rods have a yellow droplet; others have none at all. Now, it is beyond a doubt that the red rays penetrate more easily than any others into the rods with a red droplet; that blue, yellow and green rays produce relatively more impression upon the rods with yellow droplets; blue rays are probably almost entirely excluded from these two kinds of rods to affect only the more strongly the uncolored rods. It is therefore very probable that these different rods are the terminal organs of the nerves sensitive to red, to green, and to blue.

---

* We have not yet succeeded in exactly determining by experiment the three fundamental colors. In regard to red alone there is no doubt; experiments made upon certain persons affected with color-blindness prove that this color corresponds to the extreme red of the spectrum. Thomas Young chose violet as the fundamental color of the other extremity, while Maxwell found the probabilities greater in favor of blue; we cannot yet definitely decide the question.

A very analogous hypothesis has since appeared to me to be well fitted to explain, in the simplest manner, the peculiarities, just as remarkable, which the perception of musical sounds presents. We know that in the cochlea of the ear the extremities of the nerve fibres are regularly spread out beside one another, and are furnished with little elastic appendices, which are called the *fibres of corti*, which are arranged regularly beside one another, like the keys and hammers of a piano forte. I supposed that each particular nerve fibre is capable of perceiving a sound of a certain pitch, that one which makes it elastic appendix vibrate the most strongly. This is not the time to dwell upon the special characters of the sonorous sensations which have led me to propound a hypothesis whose analogy to the theory of colors of Young is evident, and which reduces to a principle, just as simple as that of Young's theory of colors, the formation of harmonics and of beats, the perception of timbres, the difference between consonance and dissonance, the formation of the musicial garnut, etc. I ought to say that the ear offers an anatomical structure much more easily reconcilable with a hypothesis of this kind; we have even succeeded lately in obtaining a direct verification of it, not in man or the other vertebrata, in whom the labyrinth of the ear is too inaccessible, but in certain marine crustacea. In these animals the appendices of the auditory organ are external; they may be observed upon the living animal in the form of articulated hairs in which the fibres of the auditory nerve terminate, and Mr. Hensen (of Kiel) has been able to show that different sounds cause sometimes one part, and sometimes another of these hairs to vibrate.

It is necessary for us to reply here to an objection which may be made to the theory of colors of Thomas Young. I have said before that, in the representation of the system of the colors upon the chromatic table, the line where the most saturated colors—those of the spectrum—are figured resembles a triangle. Now, our arguments upon the theory of three fundamental colors demand that this triangle should be absolutely rectilinear, because thus only is it possible to obtain all the colors by the mixture of the three fundamental colors situated at the angles of the triangle. But, let us not forget it, the chromatic table contains all the colors of the external world, while in the theory which occupies our attention, the question is concerning the composition of sensations. It suffices to suppose that objective lights* do not produce perfectly pure colored sensations, that is to say, that simple red light, even perfectly free from white light, does not excite solely the fibres sensative to red, but also, though feebly, the fibres sensitive to green, and still more feebly those sensitive to violet. Therefore the sensation which the purest red light produces in the eye is still not the purest sensation of red, and we may conceive of a red more saturated still than that of which any color of the external world can give us the impression.

Experiment confirms the occuracy of this supposition, and it is possible to obtain such a sensation of more saturated red. This fact is very important, not only because it overthrows an objection which may be made to the theory of Young, but also on account of the signification which it possesses in relation to sensations of color in general. To describe this experiment, it is necessary to commence by setting forth an entirely new order of phenomena.

Every nervous apparatus becomes fatigued when it is kept in activity, and this by so much the more as it acts with greater intensity and for a greater length of time. The red blood which runs in the arteries constantly renews the effete matter, and repairs the changes produced by the activity, that is to say, the fatigue. The same thing takes place in the eye, when we fatigue the retina over its whole surface by remaining, for example, some time in the open air on a clear day, it becomes insensible to a feeble light. If we then enter a dark place, at first we can see nothing; we then say we have been blinded. After some time the eye recovers itself, and finally we see well enough, to be able to read in a place which at first appeared completely dark.

In this manner general fatigue of the retina is manifested; but a partial fatigue may also be produced, when one part only has been strongly illuminated for some time. If we fix our eyes for some time upon a point of a bright object which is isolated on a dark back-ground—the fixation is necessary so that the bright image may remain immoveable upon the retina and fatigue only one accurately defined portion of its surface,—and then turn the

* [Our Author applies the term *lights* to those objects which reflect light, as well as to those which produce it. Trans.]

eyes toward a surface of a uniform dark gray tint, we see upon this surface an *accidental image* of the object previously seen; the contours have not changed but the illumination has become inverse, the dark surface appears bright, and the bright object dark, just as it would have been on a photographic negative. By carefully fixing we may obtain very finely drawn accidental images; sometimes we may even distinguish letters. The accidental image is formed as a consequence of local fatique; the parts of the retina which have been strongly illuminated perceive less clearly than the surrounding parts the light of the gray surface so that upon this perfectly uniform surface we think we see a dark spot equal in extent to that which was just before very bright.

Sheets of white paper in a good light are sufficiently luminous objects for the formation of accidental images; when we look at much more luminous objects, such as the sun or flames, it happens that during the first instants the positive accidental image, produced by the persistence of the impression, superimposes itself upon the negative image which is the effect of fatique; moreover, the different colors contained in white light produce effects differing in duration and force. As a result we then see colored accidental images, and the whole phenomenon becomes much more complicated.

Accidental images furnish an easy means of demonstrating that the intensity of the impression produced by a bright surface commences to diminish from the first seconds, and that after a minute it is generally reduced one half or three fourths. The simplest manner of trying the experiment is, to half cover a sheet of white paper with a sheet of black paper, then to fix the eyes upon a certain point of the white sheet near the border of the black one, and to quickly draw away the black sheet, after from thirty to sixty seconds, without moving the eyes. Then, at the place which was black the impression of white is produced with its first freshness. and one is astonished to see how much the impression has become enfeebled and deadened for the rest, during the little time that the experiment has lasted. It is neccessary to remark, however, that this great diminishion of apparent intensity passes completely unerceived so long as we do not remove the black paper.

We may observe still another kind of partial fatigue, relating to certain colors only.

It is sufficient for this to expose the whole or a part of the retina to the illumination of a single color for some time, that is to say from thirty seconds to five minutes. According to the theory of Young it is clear that, in this experiment, fatigue affects only one or two kinds of fibres sensitive to light, and does not act upon the others. It follows from this that if we look, for example, at the accidental image of red upon a gray surface, the uniform light of this surface can produce strongly only sensations of green and violet in that part of the retina fatigued by the red. This part is slightly affected with anerythropsy; its accidental image therefore appears bluish green, the color complementary to red.

This, finally, affords us the means of really producing upon our retina unadulterated sensations of pure and saturated colors. To obtain saturated red, for example, we have only to fatigue one part of the retina with the blueish green of the spectrum, the color complementary to red. We thus render this part of the retina blind, for the time, to the green and the violet. Let us project the accidental image thus obtained upon the red of a prismatic spectrum as pure as possible; this image then appears of an intense saturated red, and the red of the spectrum which surrounds it, and which is yet the purest red which the external world can show us, appears less saturated, because it is painted upon the unfatigued portions of the retina: it appears as if covered with a whitish fog.

Let us content ourselves with these examples; I do not wish to accumulate more details, whose exposition would involve us in innumerable descriptions of experiments.

In the presence of these facts, is it possible to suppose, as we are naturally disposed, to, that the qualities of our sensations, and particularly those of our visual sensations, are a representation of corresponding qualities of the external world. Evidenty not. The contrary has been set forth already in the law of J. Muller concerning the specific energies of the senses, a law founded on an observation of the facts. If the rays of the sun are manifested to us under the form of color, or under that of heat, it is something which does not depend upon their intimate constitution; it varies according as they excite the optic nerve fibres or the nerve fibres of the skin. A pressure upon the eyeball, a feeble electric current which traverses it, a narcotic coursing in the blood, may be perceived under the form of light as well as the rays of the sun. The profound differences which exist between the sensations of vision, of hearing, of taste, of smell and of touch, a difference as radical as the sensation of color and of sound, have between themselves no relation of resemblance nor even of dissemblance, the difference depends not at all upon the nature of the external object, but solely upon the central relations of the affected nerve. The question whether, in the domain of each sense, there exists an accord between the objective and the subjective becomes therefore secondary. The colors which the different systems of the ether waves present to us vary certainly with the wave-length of the vibrations of the ether which make an impression on the optic nerve. The system of colors naturally visible enables us to distinguish a series of differences between the different luminous mixtures; but the number of these differences is extremely limited: it is in the ratio of three to infinity. Since the most important faculty of the eye is to measure dimensions in space, and since, in this relation, its organization is much more delicate than that of the ear, we have a right to be satisfied if the eye distinguishes any qualitative differences of light, though very few relatively. The ear, which is much more richly endowed relatively to qualitative differences, is almost incapable of distinguishing anything as it relates to space. Is it not astonishing that in man who, unless he is forwarned, accords full confidence to the testimony of the senses, neither the limits of the visibility of the spectrum, nor the boundaries of the few differences of color which he is capable of perceiving are founded upon anything which has existence outside of the visual faculties? The light may have absolutely the same aspect while being completely different in all its other known physical and chemical aspects.

We find finally, that the pure elements of our sensation of colors, the sensation of the pure fundamental colors, cannot be perceived by the unfatigued natural eye only upon condition of artificially arranging affairs in suitable conditions, and that these colors exist only in the condition of subjective phenomena.

The relation which does subsist between the quality of the external light and that of the sensation may at first sight appear to be but a very little thing, but, in reality, this is perfectly sufficient for an infinite number of the most useful applications. *Identical light excites under the same conditions identical sensations. Light which under the same conditions excites differing sensations of color is not identical.* [To be Continued.]

